# The Post-Amalgam Era: Norwegian Dentists’ Experiences with Composite Resins and Repair of Defective Amalgam Restorations

**DOI:** 10.3390/ijerph13040441

**Published:** 2016-04-22

**Authors:** Simen E. Kopperud, Frode Staxrud, Ivar Espelid, Anne Bjørg Tveit

**Affiliations:** 1 Nordic Institute of Dental Materials (NIOM), Oslo 0855, Norway; 2 Faculty of Dentistry, University of Oslo, Oslo 0316, Norway; frode.staxrud@odont.uio.no (F.S.); ivar.espelid@odont.uio.no (I.E.); a.b.tveit@odont.uio.no (A.B.T.)

**Keywords:** dentistry, amalgam, composite resin, operative treatment, minimally invasive dentistry, minimal intervention dentistry

## Abstract

Amalgam was banned as a dental restorative material in Norway in 2008 due to environmental considerations. An electronic questionnaire was sent to all dentists in the member register of the Norwegian Dental Association (NTF) one year later, to evaluate dentists’ satisfaction with alternative restorative materials and to explore dentists’ treatment choices of fractured amalgam restorations. Replies were obtained from 61.3%. Composite was the preferred restorative material among 99.1% of the dentists. Secondary caries was the most commonly reported cause of failure (72.7%), followed by restoration fractures (25.1%). Longevity of Class II restorations was estimated to be ≥10 years by 45.8% of the dentists, but 71.2% expected even better longevity if the restoration was made with amalgam. Repair using composite was suggested by 24.9% of the dentists in an amalgam restoration with a fractured cusp. Repair was more often proposed among young dentists (*p* < 0.01), employees in the Public Dental Service (PDS) (*p* < 0.01) and dentists working in counties with low dentist density (*p* = 0.03). There was a tendency towards choosing minimally invasive treatment among dentists who also avoided operative treatment of early approximal lesions (*p* < 0.01). Norwegian dentists showed positive attitudes towards composite as a restorative material. Most dentists chose minimally- or medium invasive approaches when restoring fractured amalgam restorations.

## 1. Introduction

As of 1 January 2008, the use of amalgam as a dental restorative material has been banned in Norway. The ban was not directly a ban of amalgam as a restorative material, even though the Norwegian government had put pressure on dentists to reduce the use of amalgam during the preceding years [1], but rather a general ban of all mercury-containing products issued by the Norwegian Ministry of Climate and Environment due to environmental considerations [2]. In a guest editorial in the most prestigious research journal in dentistry, this decision was highly criticized [3]. In a response to the editor, however, it was claimed that “As Norway decreases its own pollution, it will decrease global mercury pollution, enhancing global health” [4]. This reflects some of the controversy associated with amalgam as a restorative material in teeth. Previous studies from Norway show that the use of amalgam was decreasing and use of composites increasing in the years preceding the ban [5] and that in absence of amalgam, composite definitively became the most preferred material for restoration of posterior teeth [6]. Nevertheless, the ban was criticized by many dentists in Norway whose general perception was that longevity of amalgam restorations was superior to that of composite. These opinions could be due to several previous cross-sectional studies showing superior longevity of amalgam compared to composite [7,8,9,10]. However, cross-sectional studies have been criticized for underestimating the longevity of newer restorative materials, due to differences in observation time such as new composite restorations in a time period where amalgam had been used for decades [11]. Thus, the assumption in the past that composites had a lower longevity than amalgam, as suggested by the referred cross-sectional studies, might not be true. Additionally, it has been suggested that the differences in longevity seen in previous studies were due to differences in the skill of placing composites, since many studies were performed in a time when dentists placed mostly amalgam restorations and few composite restorations [12]. The authors concluded that operators who are skilled in both placing amalgam and composite restorations should be able to achieve comparable longevity today.

According to modern dental philosophy, repair of defective restorations should always be considered when choosing among the available treatment options [13,14]. If a full replacement of the restorations is performed, a significant amount of tooth structure is removed and the preparation enlarged [15]. The major advantage of repair is to save tooth substance, and thus the approach is consistent with the concept of *minimal intervention dentistry* [16]. Composite restorations are considered repairable [13,17,18]. Repair of defective amalgam restorations with new amalgam has been shown successful [19,20], while repair of amalgam with composite has shown variable results [13,21]. So, how will Norwegian dentists treat defective amalgam restorations when the use of amalgam is not allowed? The present study aimed to evaluate dentists’ satisfaction and opinions on composite compared with amalgam as a restorative material, one year after the amalgam ban was issued. Further, the study aimed to explore dentists’ preference for treatment of a fractured amalgam restoration.

## 2. Material and Methods

In March 2009, a pre-coded questionnaire was sent electronically to all dentists (dental surgeons) with an e-mail address registered in the member register of the Norwegian Dental Association (Den norske tannlegeforening—NTF), using the Internet-based software QuestBack. Of the 4315 members of NTF, 3654 e-mail addresses were registered. Participation was voluntary and no remuneration was offered to the respondents. The software QuestBack was configured to send automatic reminders to all participants who did not reply within three and five weeks, respectively. Anonymity was ensured by QuestBack. The study was approved by the Norwegian Social Science Data Services (NSD) (Project number 21170).

Information was collected regarding the respondents’ sex, age, home county, type of practice and to which extent the respondent was occupied with caries diagnosis and treatment in his/her practice. Questions were asked about the use of restorative material in Class II-restorations, opinion factors related to the failure of Class II composites and general attitudes towards composites as shown in Table 1, Table 2, Table 3 and Table 4. 

**Figure 1:**
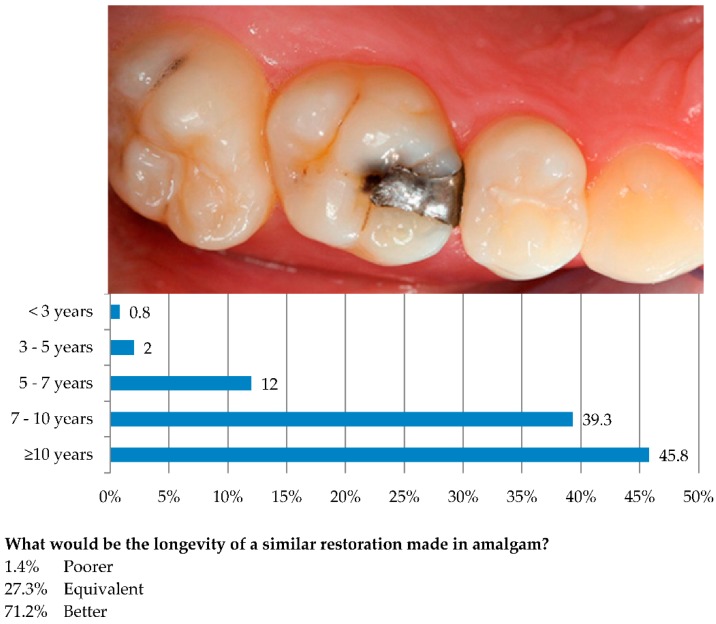
*Patient Case 1:* What longevity would you estimate if the amalgam restoration in this upper first molar was to be replaced with composite? The amalgam restoration has a small secondary caries lesion that is barely visible on x-ray. The restoration has gingival enamel in the approximal box. The patient is a 40-year-old woman with satisfactory oral hygiene, uses fluoride toothpaste and has a dental check-up every 12 months. She wants to replace the whole restoration with composite.

Two patient cases were presented to the dentists: *Patient Case 1* showed an upper first permanent molar with a small MO amalgam restoration that needed to be replaced (Figure 1). The dentists were asked what longevity they would estimate for a new restoration if the amalgam restoration was to be replaced with composite. The amalgam restoration was said to have a small secondary caries lesion that was barely visible on x-ray. The restoration had gingival enamel in the approximal box. The patient was a 40-year-old woman with satisfactory oral hygiene using fluoride toothpaste. She attended the dentist for a check-up every 12 months. She wanted to replace the whole restoration with composite. 

**Figure 2:**
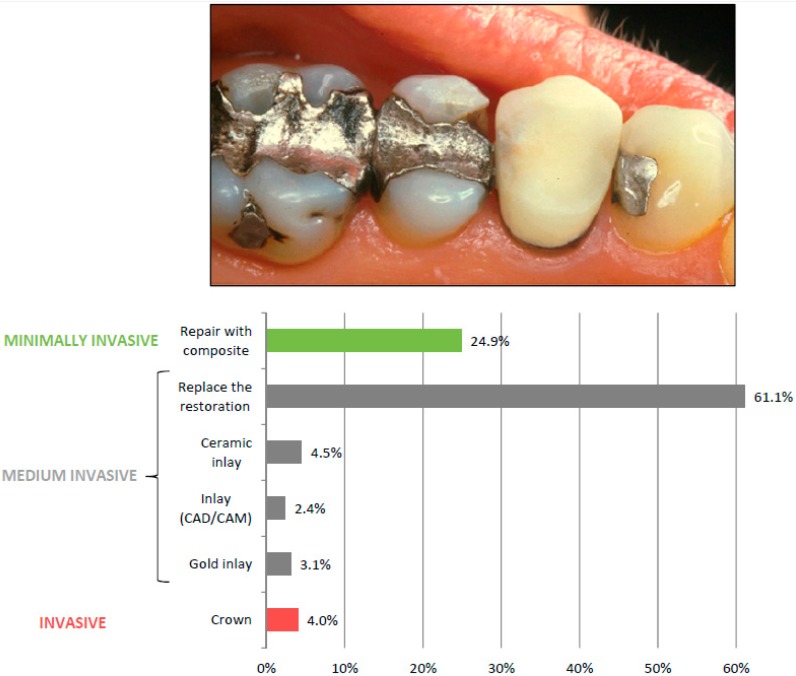
*Patient Case 2*: What is in your opinion on the best treatment for this upper second premolar? The tooth has a fractured amalgam restoration. There is no sign of secondary caries. The restoration has cervical enamel in both the approximal boxes. Radiological examination shows that the amalgam restoration has good distance to the pulp. No other pathology is noticed. The patient is a 52-year-old woman with low caries activity and normal occlusion. She has no aversion towards amalgam and there are no economical limitations regarding the choice of treatment.

*Patient Case 2* showed an upper second permanent premolar with a fractured amalgam restoration and no sign of secondary caries (Figure 2). The restoration was said to have cervical enamel in both approximal boxes. Radiological examination showed that the amalgam restoration had good distance to the pulp. No other pathology was noticed. The patient was a 52-year-old woman with low caries activity and normal bite. She had no contradictions towards amalgam and there are no economical limitations on the preferred treatment. The respondents could choose what they considered to be the best treatment from a precoded list. Their treatment decisions were grouped according to amount of tooth substance removal as either (1) Minimally invasive—Repair with composite; (2) Medium invasive—Replace the restoration with either filling or inlay; or (3) Invasive—Restore the tooth with a crown.

Statistical analyses were performed by descriptive statistics with chi-square tests and two separate logistic regression analyses with the dependent variables: “Minimally invasive treatment” and “Invasive treatment” (Figure 2). Independent variables were the dentist’ s age and gender, type of practice, mean number of decayed, missing, and filled teeth (DMFT) for 18 year olds and number of patients per dentist (dentist density) in the respondents’ respective counties of practice. Aggregated data for each of 20 Norwegian counties on the two latter variables were extracted from Statistics Norway, Dental Health [22]. Variables significant at *p* ≤ 0.2 level in the unadjusted analyses were entered into the adjusted logistic regression analysis. Collinearity was checked using the criterion Variance Inflation Factor < 5 and no independent variables were found to invalidate the analysis. Statistical analyses were performed using IBM Statistical Package for the Social Sciences (SPSS) Statistics version 20.0.0.1 (SPSS Inc., Chicago, IL, USA). A significance level of 5% was used throughout.

## 3. Results

In total, 2375 out of 3654 dentists responded after two reminders. A response rate of 61.3% was calculated according to the Standard Definitions of the American Association for Public Opinion Research [23]. Respondents 69 years of age and older (*n* = 63) and those who did not normally work with caries and filling materials (*n* = 286) were excluded from the statistical analyses, leaving a total of 2026 included respondents. The mean age of the included dentists was 46.2 years (SD 11.9), 47.1% female and 52.9% male. The distribution of age and gender of the included respondents did not differ significantly from all dentists in the NTF member register and the Norwegian Registration Authority for Health Personnel (SAFH) [6]. According to the type of practice, 690 (34.1%) of the included respondents were employed by the Public Dental Service (PDS), 1299 (64.1%) were private practitioners and 37 (1.8%) were employed elsewhere, e.g., in research or administrative work. In the member register of the NTF, 32.9% were employed by the PDS and 67.1% were registered as private practitioners.

Composite was the preferred restorative material for Class II restorations in premolars and molars among the majority of dentists (Table 1). Other restorative materials and techniques were sparsely used. Table 2, Table 3 and Table 4 show the dentists’ opinions on the reasons for failure and factors affecting the longevity of Class II composites. Secondary caries was the most commonly reported cause of failure, stated by 72.7% of the dentists to be “Often” or “Always” the cause for replacement. Restoration fractures and poor approximal contact were the second and third most common reasons for replacing composites, reported “Often” or “Always” by 25.1% and 23.4% of the dentists, respectively. Moisture control during placement of composite restorations (58.9%) and the caries activity of the patient (52.1%) were considered to be very significant factors for the longevity. In *Patient Case 1* (Figure 1), almost half of the dentists (45.8%) estimated the longevity to be ≥10 years for an MO composite replacement of a defective amalgam restoration, 39.3% estimated 7–10 years longevity and 14.8% estimated longevity of less than 7 years. The majority of the dentists (71.2%) expected an even better longevity if the restoration was made in amalgam, 27.3% anticipated equivalent longevity, while 1.4% estimated a poorer longevity of an amalgam *versus* a composite restoration. Among the dentists who anticipated better longevity of an amalgam restoration compared with composite in *Patient Case 1*, more were females, dentists in the two youngest age groups (<48 years) and those employed in the PDS (*p* < 0.01). Only 34.4% of these dentists estimated the longevity of a composite restoration in *Patient Case 1* to be ≥10 years, compared with 73.4% of dentists who expected equivalent or poorer longevity of an amalgam restoration (*p* < 0.01). In Table 4 it is shown that 74.0% of all dentists agreed (either “Agreed” or “Totally agreed”) with the statement that: «Composite is a good alternative to amalgam». Concerning *Patient Case 1*, 66.8% the dentists who expressed that they anticipated a restoration in amalgam to have better longevity than composite, agreed with the statement above. When it comes to those who expressed the opinion that amalgam in this case had equivalent or poorer longevity compared to composite, 91.9% agreed with the statement. 

The dentists’ treatment decisions for *Patient Case 2* are illustrated in Figure 2. The treatment decisions were grouped according to amount of tooth substance removal as either (1) Minimally invasive—Repair with composite (24.9%, *n* = 502); (2) Medium invasive—Replace the restoration with either filling or inlay (71.1%, *n* = 1432) or (3) Invasive—Restore the tooth with a crown (4.0%, *n* = 80). Their choices of treatment were examined by use of logistic regression analyses. Minimally invasive treatment was significantly more often proposed among young and female dentists, employees in the PDS and dentists working in counties with a low dentist density (unadjusted analyses). When adjusting for all other variables, dentists’ gender did not reach significance, while all other variables remained significant (Table 5). Invasive treatment (crown) was significantly more often proposed by male dentists and dentists working in counties with high dentist density (unadjusted analyses). Both variables remained significant when adjusting for all variables (Table 5).

Combining the respondents’ answers to *Patient Case 1* and *Case 2* showed that dentists who chose a minimally invasive approach in fact had a more pessimistic view on the longevity of composite restorations compared with dentists who chose a medium invasive or invasive approach. A significantly smaller amount of the dentists who chose a minimally invasive approach in *Patient Case 2* estimated the longevity of a composite restoration in *Patient Case 1* to be ≥10 years (36.1%), compared with dentists choosing an either medium invasive or invasive approach (48.8%) (*p* < 0.01). 

Also, significantly more dentists choosing a minimally invasive approach expected the longevity to be longer if the restoration was made in amalgam (77.6%), compared with dentists choosing an either medium invasive or invasive approach (69.1%) (*p* < 0.01).

The dentists’ thresholds for instigating operative treatment of approximal caries lesions have been explored in a previous paper [6]. Table 6 shows a cross tabulation of the treatment decisions for *Patient Case 2* and the decision to operatively treat approximal caries. There was a significant tendency towards choosing minimally invasive treatment in *Patient Case 2* among dentists who also avoided operative treatment of early approximal primary caries lesions (*p* < 0.01). Likewise, dentists who chose an invasive treatment strategy in *Patient Case 2* also treated early stages of approximal caries more often (*p* < 0.01).

## 4. Discussion

The Norwegian Dental Association (NTF) estimates that 90%–95% of all practising dentists in Norway are registered members. The relatively high response rate (61.3%) and the matching age distribution of the respondents are consistent with our sample being representative of the members of NTF and all authorized dentists in Norway. Our response rate was considered satisfactory, and in the high-end of what has been achieved in similar questionnaire studies elsewhere [24,25,26,27,28].

In Scandinavia, use of amalgam is more or less banned; in Norway since 2008 [2] and in Sweden since 2009 [29] with some exceptions. The use of amalgam is still allowed in Denmark, but the government has put strong restrictions in place. The Minamata Convention on Mercury is a global treaty to protect human health and the environment from the adverse effects of mercury. The treaty is at present signed by 128 countries [30] and its repercussions call for a phase-out of dental amalgam [31]. Thus, a ban on amalgam could also be forthcoming in other countries and experiences among Norwegian dentists after the ban could be a valuable contribution to a foregoing debate on this subject. Although the present data was collected in 2009 and our conclusions are likely to be outdated in a Norwegian setting, the findings may have high clinical relevance in other societies where use of amalgam is still allowed and being phased-out. Our results reflect decision making on restoration replacement in a population of dentists that are not using amalgam anymore. In the UK, Lynch and Wilson have already used Norway as an example on how to manage a phase-down and eventually ban of amalgam [32]. The present study could be considered an important follow-up on this matter, providing information on how the dentists cope with a ban of amalgam.

Table 1 shows the dentists’ preferred restorative material in a MOD-cavity. It demonstrates that composite has become the dominating material of choice in Norway one year after the amalgam ban. Almost all dentists (99%) stated that they “Often” or “Always” used composite when restoring a MOD-cavity due to primary caries being confined to the outer half of dentin. Similar trends have been found in other Norwegian studies [5,6]. International studies show similar tendencies; a study on trends in dental treatment in the USA showed that patients received approximately 50% fewer amalgam fillings in 2007 compared with 1992, while the rise in use of resin-based composite restorations was equivalent [33]. In other countries, the use of amalgam has also decreased rapidly [12,24,26,33,34,35,36,37]. More than fifty percent of the dentists in our questionnaire study stated that they “Never” used other materials than composite. This is in accordance with a recent practice-based study showing that the overall use of other materials than amalgam and composite was only 5% for both U.S. and Scandinavian dentists, when placing restorations in premolars and molars [38]. 

The general opinion that secondary caries and restoration fracture are the most common reasons for failure of composites (Table 2) is supported by evidence from the literature. A review of studies conducted in the 1990s on the longevity of dental restorations reported that secondary caries was the reason for replacement in 33%–65% of failed composite restorations [39]. Studies published later have reported similar rates: 25% [40], 38% [12], 52% [41], 57% [42] 58% [43] and 88% [44]. In a recent review on the longevity of posterior composite restorations, secondary caries and fracture of restoration are considered the two main reasons for failure [45]. The dentists in our questionnaire study considered moisture control during placement of composite restorations (58.9%) and the caries activity of the patient (52.1%) to be very important factors for the longevity of composites (Table 3). The findings are consistent with replies shown in Table 4. Both these variables could be related to development of secondary caries. Nevertheless, the lack of standardized diagnostic criteria for marginal failure could cause over-registration of secondary caries [46,47]. Crevices and ditched margins in which the explorer sticks, and marginal colour changes, could be wrongly diagnosed as secondary caries [47,48,49]. 

In Table 4, 58.5% of the dentists either “Agree” or “Totally agree” that secondary caries is more commonly seen in composite restorations compared to amalgam. This is in accordance with findings in a questionnaire study on Finnish dentists’ perceptions on the reasons for replacement of restorations [50]. This perception is clinically established in the literature; in a retrospective clinical study by Kuper *et al.*, composite restorations developed secondary caries twice as often as amalgam restorations [43]. Similar results have also been shown in three earlier RCT studies [41,44,51]. Post-operative pain or sensitivity were reported “Never” or “Seldom” to be the reason for failure by 50.5% of the dentists and additional 43.8% reported only “Sometimes”. This corresponds well with the conclusion in a review by Hickel *et al.* that the problem with post-operative hypersensitivity was decreasing [52]. Allergic reactions were reported “Never” or “Seldom” to be the reason for failure by 93.8% of the dentists. The prevalence of adverse reactions to composites in Norway is reported to be generally low. From 1993, the Norwegian Dental Biomaterials Adverse Reaction Unit has operated a national reporting procedure concerning suspected biologic adverse reactions experienced in relation to treatment with dental biomaterials, but during the twenty years from 1993 to 2013, only about 2100 reports have been received. In 2013, 28% of the reports were related to composites and cements, a percentage that has remained relatively stable over the years following the amalgam ban [53].

In general, the dentists’ replies in *Patient Case 1* indicate a positive view on the longevity of restorations in a low-risk patient. Nearly half the dentists (45.8%) estimated the longevity to be more than ten years, while only 3% of the dentists estimated the longevity to be less than five years. The positive trend is consistent with the presented views in Table 4 where 74% of all dentists either “Agreed” or “Totally agreed” with the statement that composite is a good alternative to amalgam. Nevertheless, the fact that 71.2% of the dentists expected longevity to be better if the restoration was made with amalgam distorts the picture. These dentists were found significantly more often to be young, female and employed by the PDS. This diverges partly from what was previously found in a practice-based clinical study, where the dentists who preferred amalgam in Class II restorations were identified as being male and the patients to have high caries experience [5].

Logistic regression analyses revealed that minimally invasive treatment (repair) in *Patient Case 2* was suggested more often by dentists working in counties with low dentist density, while invasive treatment (crown) was suggested more often by dentists working in counties with high dentist density. These findings indicate that dentist remuneration affects the treatment decision. Repair is a rapid and cheap alternative that can be preferred among dentists who have many patients attending their dental clinic, while a crown generally produces more work at a considerably higher cost, which could be beneficial for dentists with few patients attending their dental clinic. This idea is supported by the finding that more dentists employed in the PDS also chose repair, since they often have high workload and, in many cases, a fixed salary.

Dentists choosing minimally invasive treatment in *Patient Case 2*, tended only to treat advanced stages of approximal caries lesions operatively (Table 6). This is supported by findings in a study by Heaven *et al*. who found that dentists who recommended restorative treatment of primary occlusal caries and approximal caries at a more advanced stage were significantly more likely to recommend repair instead of replacement of a defective restoration [27]. 

## 5. Conclusions

Norwegian dentists showed positive attitudes towards composite as a restorative material one year after amalgam was banned. This has been confirmed by a later report by The Norwegian Climate and Pollution Agency which indicates that “dental personnel and patients generally are satisfied with the alternatives to dental amalgam” [54]. Most dentists choose minimally invasive- or medium invasive approaches when restoring fractured amalgam restorations. Dentists choosing minimally invasive treatments also avoid operative treatment of early approximal lesions.

## Figures and Tables

**Table 1:** Which restorative materials do you use when restoring a MOD-cavity due to primary caries confined to the outer half of dentin (%)? The question is related to premolars and molars in adult patients.

Restorative Material	Never	Seldom	Sometimes	Often	Always	*n*
Composite	0.1	0.1	0.6	36.8	62.3	2019
Compomer	76.9	14.6	6.3	1.9	0.3	1560
GIC conventional	60.6	26.6	11.3	1.5	-	1607
GIC resin-modified	60.0	27.6	11.5	0.8	0.1	1602
Composite inlay	90.6	7.5	1.8	0.1	-	1591
Ceramic inlay	56.3	31.8	10.8	1.1	0.1	1617
Ceramic inlay (CAD/CAM)	85.6	9.2	4.0	1.2	0.1	1592
Gold inlay	52.5	36.7	10.3	0.4	0.1	1662

MOD: Mesial-occlusal-distal, GIC: Glass ionomer cement; CAD/CAM: Computer-Aided Design/Computer-Aided Manufacturing.

**Table 2:** What is in your opinion the cause when Class II composite restorations in permanent premolars and molars need replacement (%)?

Reasons for Replacement	Never	Seldom	Sometimes	Often	Always	*n*
Secondary caries	0.1	1.8	25.4	70.6	2.1	2006
Restoration fracture	0.8	23.5	50.8	24.3	0.8	2001
Poor approximal contact	0.4	12.6	63.5	22.7	0.7	2006
Marginal gaps	1.1	29.7	53.3	15.4	0.5	2008
Tooth fracture	1.6	34.2	48.9	14.7	0.6	2009
Marginal defects	1.3	32.5	52.2	13.8	0.2	2008
Lost restoration	3.4	46.6	39.0	9.9	1.0	2010
Restoration wear	4.3	47.3	40.0	8.1	0.3	2008
Marginal discoloration	8.7	47.8	36.3	7.1	0.1	1992
Pain/sensitivity	2.2	48.3	43.8	5.4	0.3	2005
Poor aesthetics	7.4	55.6	33.6	3.4	0.0	2003
Restoration deficiency	5.0	58.4	33.7	2.5	0.4	2009
Porosities	8.8	62.2	27.0	1.8	0.2	2010
Overhang	9.7	63.8	24.8	1.6	0.2	2001
Allergic reactions	45.1	52.7	1.1	0.2	0.9	1999

**Table 3:** To which extent do you think the following factors have significance for the longevity of a Class II composite restoration (%)?

Factors Relevant for Longevity	Do Not Know	None	Minor	Medium	High	Very High	*n*
Moisture control	0.1	0.1	0.7	4.8	35.4	58.9	2009
High caries activity	-	-	0.6	5.3	41.9	52.1	2005
Poor oral hygiene	-	-	1.1	11.5	46.2	41.0	2003
Poor matrix technique	0.3	0.1	1.0	11.6	50.4	36.4	2001
Patient cooperation	0.5	1.0	11.5	30	39.6	17.5	1999
Cavity design	0.1	0.3	11.1	37.6	40.8	10.1	2006
Hard bite (patient)	0.6	0.2	11.3	42.9	35.7	9.4	1992
Following manufacturer’s instructions	0.2	0.5	7.9	51.0	40.3	0.2	2008
Dentist’ s experience	0.6	1.5	11.0	54.4	32.4	0.6	2010
Type of adhesive	1.9	1.0	26.8	43.7	21.8	4.8	2008
Type of composite	1.8	2.2	37.4	44.3	12.0	2.3	2008

**Table 4:** Relate to the following statements regarding composite restorations (%).

Statements	Do Not Know	Totally Disagree	Disagree	Neutral	Agree	Totally Agree	*n*
Moisture control is the most important factor to achieve successful restorations	0.1	0.5	2.9	13.6	47.7	35.3	2014
Composite is a good alternative to amalgam	0.3	0.5	6.2	18.9	44.4	29.6	2016
Secondary caries is more commonly seen in composite restorations compared with amalgams	1.9	2.4	13.8	23.4	40.1	18.4	2003
Composite is not suitable in patients with high caries activity	0.4	2.8	31.5	35.8	23.7	5.7	2009
Lining is not necessary in deep composite restorations	0.2	13.2	40.9	17.9	21.6	6.2	2002
I often experience that my composite restorations need replacement	0.4	6.0	43.4	30.3	17.8	2.1	2010
Composite is not suitable in patients with poor oral hygiene	0.3	7.3	39.2	35.2	15.7	2.2	2003
Composite is not suitable in patients with a hard bite	0.4	6.0	48.5	34.4	9.9	0.8	2002
Composite is only suitable in small cavities	0.2	24.0	57.6	12.8	3.8	1.7	2015
Composite is not suitable in molars	-	39.0	53.9	5.6	1.1	0.3	2001

**Table 5:** Variables related to the dentists choice of a minimally invasive and invasive treatment approach in *Patient Case 2.*

Independent Variables	% (*n*)	Minimal Invasive Treatment						Invasive Treatment					
		Unadjusted			Adjusted			Unadjusted			Adjusted		
		OR	95% CI	*p*-Value	OR	95% CI	*p*-Value	OR	95% CI	*p*-Value	OR	95% CI	*p*-Value
**Dentist’s Age**													
≥35 years	27.5 (557)	-	-	-	-	-	-	-	-	-	-	-	-
36–47 years	27.0 (548)	0.73	0.56–0.95	0.02	0.80	0.61–1.05	0.10	0.76	0.42–0.95	1.41	0.71	0.39–1.32	0.28
≥48 years	45.5 (921)	0.51	0.40–0.65	<0.01	0.54	0.42–0.69	<0.01	0.87	0.51–0.65	1.46	0.74	0.43–1.27	0.28
**Dentist’s Gender**													
Female	48.1 (974)	-	-	-	-	-	-	-	-	-	-	-	-
Male	51.9 (1052)	0.62	0.51–0.76	<0.01	0.82	0.65–1.02	0.07	1.97	1.23–3.18	<0.01	2.06	1.24–3.42	2.06
**Practice Type**													
Private Practice	64.1 (1299)	-	-	-	-	-	-	-	-	-	-	-	-
Public Dental Service	34.1 (690)	2.36	1.92–2.91	<0.01	2.19	1.76–2.72	<0.01	0.62	0.37–1.03	0.07	0.79	0.46–1.35	0.38
Other	1.8 (37)	1.37	0.64–2.94	0.42	1.52	0.70–3.30	0.29						
**DMFT in County**													
Continuous Variable	100 (2026)	1.03	0.88–1.19	0.73				0.89	0.63–1.24	0.49	-	-	-
**Number of Patients per Dentist in County**													
Continuous Variable	100 (2026)	1.01	1.00–1.01	0.01	1.01	1.00–1.01	0.03	1.01	1.00–1.01	0.01	1.01	1.00–1.01	0.01

**Table 6:** Cross tabulation of the decision on how to treat *Patient Case 2* and threshold for operative treatment of approximal primary caries. Most dentists choosing minimally invasive treatment in *Patient Case 2* only treated advanced stages of approximal caries operatively.

Treatment decisions in *Patient Case 2*	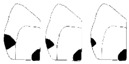	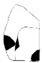	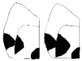
	Enamel Caries	Caries in Outer Third of Dentin	Caries in Middle and Inner Third of Dentin
Minimally invasive (repair) (*n* = 501)	4.8%	50.5%	44.7%
Medium invasive (restoration/inlay) (*n* = 1428)	7.1%	58.8%	34.1%
Invasive (crown) (*n* = 79)	10.1%	63.3%	26.6%

## Abbreviations

The following abbreviations are used in this manuscript:

CAD/CAM: Computer-Aided Design/Computer-Aided Manufacturing
DMFT: Decayed, missing and filled teeth
GIC: Glass ionomer cement
MO: Mesial-occlusal
MOD: Mesial-occlusal-distal
NSD: The Norwegian Social Science Data Services
NTF: The Norwegian Dental Association
PDS: The Public Dental Service
SAFH: The Norwegian Registration Authority for Health Personnel
